# Conversational Agent for Healthy Lifestyle Behavior Change: Web-Based Feasibility Study

**DOI:** 10.2196/27956

**Published:** 2021-12-03

**Authors:** Dhakshenya Ardhithy Dhinagaran, Thirunavukkarasu Sathish, AiJia Soong, Yin-Leng Theng, James Best, Lorainne Tudor Car

**Affiliations:** 1 Lee Kong Chian School of Medicine Nanyang Technological University, Singapore Singapore Singapore; 2 Population Health Research Institute McMaster University Hamilton, ON Canada; 3 Centre for Population Health Sciences Lee Kong Chian School of Medicine Nanyang Technological University Singapore Singapore; 4 Centre for Healthy and Sustainable Cities Nanyang Technological University, Singapore Singapore Singapore; 5 Department of Primary Care and Public Health School of Public Health, Imperial College London London United Kingdom

**Keywords:** chatbot, conversational agents, behavior change, healthy lifestyle behavior change, pilot study, feasibility trial, usability, acceptability, preliminary efficacy, mobile phone

## Abstract

**Background:**

The rising incidence of chronic diseases is a growing concern, especially in Singapore, which is one of the high-income countries with the highest prevalence of diabetes. Interventions that promote healthy lifestyle behavior changes have been proven to be effective in reducing the progression of prediabetes to diabetes, but their in-person delivery may not be feasible on a large scale. Novel technologies such as conversational agents are a potential alternative for delivering behavioral interventions that promote healthy lifestyle behavior changes to the public.

**Objective:**

The aim of this study is to assess the feasibility and acceptability of using a conversational agent promoting healthy lifestyle behavior changes in the general population in Singapore.

**Methods:**

We performed a web-based, single-arm feasibility study. The participants were recruited through Facebook over 4 weeks. The Facebook Messenger conversational agent was used to deliver the intervention. The conversations focused on diet, exercise, sleep, and stress and aimed to promote healthy lifestyle behavior changes and improve the participants’ knowledge of diabetes. Messages were sent to the participants four times a week (once for each of the 4 topics of focus) for 4 weeks. We assessed the feasibility of recruitment, defined as at least 75% (150/200) of our target sample of 200 participants in 4 weeks, as well as retention, defined as 33% (66/200) of the recruited sample completing the study. We also assessed the participants’ satisfaction with, and usability of, the conversational agent. In addition, we performed baseline and follow-up assessments of quality of life, diabetes knowledge and risk perception, diet, exercise, sleep, and stress.

**Results:**

We recruited 37.5% (75/200) of the target sample size in 1 month. Of the 75 eligible participants, 60 (80%) provided digital informed consent and completed baseline assessments. Of these 60 participants, 56 (93%) followed the study through till completion. Retention was high at 93% (56/60), along with engagement, denoted by 50% (30/60) of the participants communicating with the conversational agent at each interaction. Acceptability, usability, and satisfaction were generally high. Preliminary efficacy of the intervention showed no definitive improvements in health-related behavior.

**Conclusions:**

The delivery of a conversational agent for healthy lifestyle behavior change through Facebook Messenger was feasible and acceptable. We were unable to recruit our planned sample solely using the free options in Facebook. However, participant retention and conversational agent engagement rates were high. Our findings provide important insights to inform the design of a future randomized controlled trial.

## Introduction

### Background

In recent years, there has been a notable increase in the incidence of chronic disease, especially among the younger population [1]. These chronic diseases include obesity and type 2 diabetes [1]. In terms of diabetes prevalence, Singapore, with 600,000 adults living with diabetes, ranks second among high-income countries, and obesity levels are also on the rise [2]. In addition, 34% of the men and 39% of the women in Singapore do not reach the weekly target of 150 minutes of moderate-intensity activity per week, leaving people at higher risk of developing diseases such as diabetes [3]. People with diabetes live shorter lives—by at least 10 years—and have a lower quality of life (QoL) than those without diabetes [4,5]. Prediabetes, a precursor to diabetes, affected 15.5% of the Singaporean adults in 2010 (ie, 1 in 7), and this figure is estimated to increase to 24.9% by 2035 [6]. Prediabetes increases the risk of heart disease, and if untreated, over time, most people with prediabetes transition to diabetes [7]. Research has shown that lifestyle interventions (such as increasing physical activity and eating a healthy diet) delivered by trained health care professionals can help to promote healthy weight loss [8-10]. In addition, lifestyle change through high-risk and population-based approaches were also directly associated with a reduction in the incidence of type 2 diabetes, accentuating the efficacy of healthy lifestyle behavior change for diabetes prevention [8].

Achieving healthy lifestyle behavior changes independently can be challenging, and support from experts such as dietitians or exercise physiologists has been shown to be more effective [8]. However, access to experts at a population level may not be feasible or affordable. A potentially more accessible alternative to in-person support and supervision could be novel digital health interventions such as conversational agents. Conversational agents, or chatbots, are computer programs designed to mimic human-to-human conversations in the form of either text messaging or verbal discourse [11]. The heightened accessibility, personalization, and efficiency that conversational agents offer highlight the potential for conversational agents to improve patient care [11-13]. Conversational agents enable 2-way communication, and their text- or speech-based method of communication makes them suitable for a variety of target populations, ranging from young children to older people. The application of conversational agents in health care is gaining traction in a number of medical fields, including health care service provision, chronic disease management, and patient education [14]. They can be delivered through a variety of means: messaging apps, individual apps, or even standalone devices [14].

Singapore is a technologically savvy country, and citizens avidly use messaging apps. In addition, Singapore’s ministry of health has proposed the increasing use of conversational agents in health care in tackling issues such as the rising chronic disease burden and the aging population [15] that can lead to more primary care appointments. The ministry envisions a near future where a conversational agent can collect a patient’s history from them before their consultation, streamlining the primary care visit and thus cutting down waiting times. This makes Singapore an ideal place for the evaluation of novel mobile health interventions such as conversational agents. Moreover, health programs delivered over the internet have shown success, as exemplified by web-based interventions for smoking [16], alcohol intake [17], sexual health [18], cancer screening [19], physical activity [20], and diet modifications [21]. The ubiquity of the internet makes these programs easily accessible to a diverse group.

### Objective

The evidence for the use of conversational agents for healthy lifestyle behavior change from trials is limited. The feasibility and acceptability of implementing and evaluating the use of novel interventions are essential for informing potential future trials. Correspondingly, we aim to assess the feasibility, acceptability, and preliminary efficacy of the use of conversational agents for healthy lifestyle behavior changes in the general population in Singapore.

## Methods

### Approval and Consent

This web-based single-arm feasibility study was approved by the Nanyang Technological University Ethics Committee (IRB-2018-11-032). All participants signed their digital informed consent before embarking on the study.

### Participants

Participants were eligible if they were aged above 21 years, were Singapore citizens or permanent residents, owned a smartphone, and had a Facebook Messenger (Facebook, Inc) account. Prospective participants were excluded if they were pregnant or had any of the following conditions: cancer, chronic liver disease, chronic kidney disease, a neurodegenerative condition, heart disease, stroke, a physical disability, hypertension, or a condition that does not allow for regular physical activity. Eligibility was confirmed by having participants complete an eligibility questionnaire, after which they were asked to provide informed consent on a digital form sent to them through email.

### Recruitment

The participants were recruited on the web through Facebook in August-September 2019. A digital poster listing the study aims and eligibility criteria was uploaded on relevant Facebook pages focused on healthy living, such as *Singapore fitness and health community* and *Singapore healthy cooking*. In addition, we used snowballing in our recruitment; therefore, participants were also procured through redistribution of our study poster through messaging apps or through word of mouth.

### Intervention

The conversational agent was designed to be used on Facebook Messenger using a free web-based tool, *Chatfuel* [22]. A research associate (AS) and a PhD student (DAD) developed the script for the program and performed the input. The intervention focused on diabetes and prediabetes knowledge, diet, physical activity, sleep, and stress management. These were the topics of focus identified in other diabetes prevention programs targeting lifestyle change [23-25].

**Figure 1:**
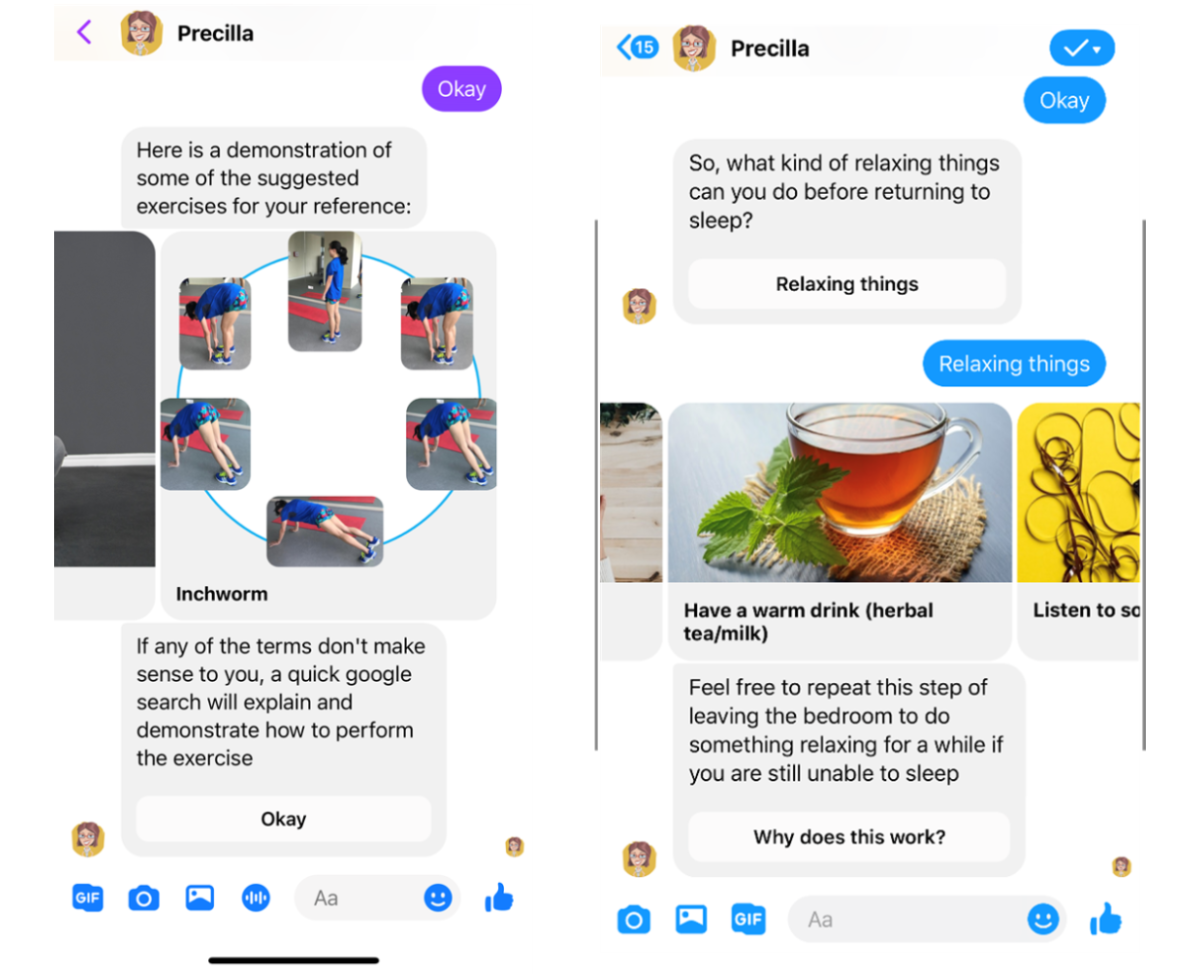
A representation of what the interactions between Precilla and users entailed.

The content was informed by existing evidence-based sources of information, including clinical guidelines and systematic reviews. Advice on improving sleep quality was generated from published evidence reporting on techniques and successful interventions for sleep disorders [26]. The domain on stress was informed by distance learning–based stress management techniques identified from a review of existing studies that described methods to reduce stress and improve health [27]. Pertinent nutritional advice for individuals with prediabetes was obtained from authenticated government health portals and other validated health and nutrition webpages [28]. The collated advice was then compartmentalized into themes, which translated into the topic of focus for each interaction between conversational agent and user (Multimedia Appendix 1 [8,26,27,29-53]). The content for the section on physical activity was informed by advice on the recommended duration and intensity of exercise from Singapore’s Health Promotion Board [28]. Validated fitness routines were then presented as part of the conversation as methods to achieve the necessary level of fitness [29]. Examples of conversational exchanges between the conversational agent and users are presented in Figure 1.

We also followed the Capability, Opportunity, Motivation, Behavior model of behavior to guide the development of the intervention [54]. This model posits that to exhibit a particular behavior (B), the participant must be physically and psychologically capable (C), have sufficient social and physical opportunities (O) to perform the behavior, and must have the desire or need to do so—motivation (M). This was important in determining the inclusion criteria for the intervention (eg, physically fit with no comorbidities) and in conversation designing.

The content was mostly presented in the form of text, supplemented with some images to make the conversational agent more engaging and to enhance the user experience. These images were obtained from free-to-use sources or photographs taken by our study team members. To further contribute to a positive user experience that mimicked human interaction, the conversational agent was given a name, *Precilla*, and it displayed human-like characteristics such as in the tone of speech, profile picture, and through using the *typing* function for messages. From here onward, we use *Precilla* to refer to the conversational agent used in our pilot study.

The intervention was scheduled to last for 4 weeks, whereby participants would receive 4 messages per week, (every other day), 1 for each topic (diet, exercise, sleep, and stress). A sample of the dialog tree is presented in Multimedia Appendix 2.

### Procedure

**Figure 2:**
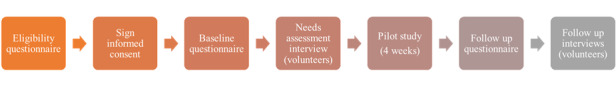
Study workflow.

An overview of the study workflow is demonstrated in Figure 2. Interested individuals were first required to complete an eligibility questionnaire. The eligible participants then provided informed consent, after which they were requested to complete a baseline questionnaire on a web-based tool, *REDCap* (Research Electronic Data Capture; Vanderbilt University) [55]. The questionnaire obtained general information regarding demographics as well as data on diet, physical activity, sleep, and stress.

The actual pilot study lasted for 4 weeks. Upon completion of the piloting period, the participants were required to complete a follow-up questionnaire that contained all the details outlined in the baseline questionnaire, with some additional questions on conversational agent usability and their overall satisfaction with the study. Interested participants also had an opportunity to take part in a follow-up interview to share their views on the conversational agent, their experience while taking part in this web-based study, as well as their thoughts on points of improvement.

### Outcomes

#### Primary Outcomes

The primary outcomes of interest for this study were the feasibility of recruitment and retention of participants, acceptability of the intervention, and participant engagement with the intervention.

#### Feasibility of Recruitment and Retention

Feasibility in this study was determined by recruitment and retention. Feasibility of recruitment was defined as the ability of the researchers to recruit at least 75% (150/200) of the target sample on the web using Facebook within a 1-month period. Feasibility of retaining participants was defined as at least 32.7% (49/150) of the recruited participants completing the study.

#### Acceptability of the Intervention

The acceptability of Precilla was measured through questions on usability and satisfaction in the follow-up questionnaire. Questions were asked on the participants’ overall satisfaction with Precilla, their likelihood of using Precilla again and recommending it to others, as well as the impact of the interactions on their health.

The usability questionnaire was split into 2 sections; the questions in section 1 related to how participants perceived the content of Precilla’s input. They were required to provide a rating for each statement from the following options: strongly agree, agree, neutral, disagree, strongly disagree. Questions were asked on ease of use, enjoyment, long-term use of Precilla, and language, as well as motivation to make healthier food choices, exercise, change sleeping habits, and better manage stress.

The questions in section 2 were concerned with the usability of Precilla and its associated delivery methods. The participants were provided with the same options to choose from as in section 1, and they were asked to share their opinions on the mode of communication (buttons, text, images, etc), the suitability of Facebook Messenger as a channel of communication, the number of messages sent, the timing of messages, and Precilla’s personality.

#### Participants’ Engagement

A further measure of acceptability of the intervention was the participants’ engagement with Precilla. Data on this aspect was collected manually by analyzing individual conversations between Precilla and the participants on Facebook Messenger. This involved noting down the duration of interactions and counting the number of complete, incomplete, and nil interactions. Immediate responses were defined as interactions made by the user within an hour of message receipt. We also collected data directly from Chatfuel analytics, such as user retention, free text typed by users (such as questions or random utterances), and the total number of attempted interactions with Precilla. In addition, the technological savviness of the study population was gauged by asking the participants to rate their own technological competency on a scale from 1 to 10.

#### Secondary Outcomes

Our secondary outcomes were related to the efficacy of the intervention with regard to the participants’ QoL, diabetes knowledge, diet, physical activity, sleep, and stress over 4 weeks.

QoL was measured using the short form version of the QoL Enjoyment and Satisfaction Questionnaire [56]. The questionnaire comprises 14 items that are rated on a 5-point scale that indicates the degree of enjoyment or satisfaction experienced during the past week. The total score for the 14 items, which cover the topics of work, social life, health, and overall well-being, ranges from 14 to 70. A percentage of the maximum score is also reported; for example, if a participant scores 20, the percentage—29% (20/70)—is also reported. The last 2 questions on medication adherence and overall life satisfaction were reported as percentages (Multimedia Appendix 3).

The knowledge questionnaire had 3 separate sections. Section 1 was concerned with participants’ knowledge about healthy living, prediabetes, and diabetes; section 2 asked for their perceptions on how healthy their lifestyle is and the likelihood of their developing diabetes; section 3 tested their knowledge of risk factors for type 2 diabetes. The questionnaire was derived from an adaptation of questions presented by the Michigan Diabetes Research Centre [57]. The questionnaire was adapted such that, in section 1, questions regarding the definitions of diabetes and prediabetes were added to test participants’ knowledge of these conditions. In section 2, the wording of some questions about participants’ efforts to make healthy lifestyle behavior changes in the past year was adapted to obtain more detail in their responses from just yes or no to a scaled format. In section 3, some risk factors that were not applicable to the Singaporean context were removed (eg, being Asian American, Hispanic, or African American).

The participants’ diets were assessed based on an adaptation of the Food Frequency Questionnaire where questions were asked on the frequency of daily intake of vegetables, fruits, fried foods, and sweetened drinks [58]. Physical activity was assessed using the International Physical Activity Questionnaire (IPAQ), which was adapted specifically for use in this study [59]. Questions were asked on the intensity of exercise (vigorous, moderate, or light), the frequency of physical activity (in days per week), and the length of each session (in minutes). Only sessions lasting at least 10 minutes qualified as physical activity. In line with IPAQ scoring, a metabolic equivalent of task (MET) score was calculated. The MET score represented the amount of energy expended when carrying out physical activity. For consistency, walking was given a score of 3.3 METs, moderate physical activity 4 METs, and vigorous physical activity 8 METs. To calculate MET minutes per week, the MET value was multiplied by the minutes for which the activity was carried out and again by the number of days in the week that the activity was undertaken. As some participants provided a range for their responses, for consistency, a mean value was used for the calculation. For example, for number of days, that is, 1-3 days, the mean was calculated as 2. For session length, that is, 10-20 minutes, the mean was calculated as 15.

A MET score of 600 METs per week indicated that an individual was moderately physically active, whereas 1500 METs per week indicated a high level of physical activity. Any score that did not qualify as moderate or high was considered an indication of a low level of physical activity.

Sleep scoring was done using the Pittsburgh Sleep Quality Index (PSQI) questionnaire [60]. A score of 0 indicated excellent sleep quality, whereas 10 indicated severely poor sleep quality. Poor sleep quality was defined as participants with a global PSQI score higher than 5. The stress level of the participants was gauged using the Perceived Stress Scale, where it was possible to receive a score between 0 and 40 [61]. Scores ranging from 0 to 13 were considered low perceived stress, from 14 to 26 moderate perceived stress, and from 27 to 40 high perceived stress.

### Data Analysis

Data analysis was conducted on the data collected at baseline and follow-up (Table 1). The participant responses on satisfaction and usability were presented as percentages of each option on a Likert scale (eg, strongly agree, disagree, much better than before, or neither better nor worse). The participant outcomes for QoL, knowledge, physical activity, sleep, and stress were all presented as pre- and postscores using the individualized scoring system of each questionnaire.

**Table 1:** Overall summary of results (N=60).

		Baseline values (n=60)	Follow-up (4 weeks) values (n=56)
**Knowledge, mean (SD; range)**			
	Section 1	7 (1.37; 6-18)	6 (0.93; 6-18)
	Section 2	28 (2.7; 12-48)	27 (3.5; 12-48)
	Section 3	10 (4.1; 8-32)	9 (2.2; 8-32)
Sleep, mean (SD)		4 (2.36)	4 (2.45)
Stress, mean (SD)		17 (5.13)	16 (5.10)
Physical activity (MET^a^ score), mean (SD)		1080 (816)	1075 (872)
**QoL^b^**			
	14-item score, mean (SD)	54 (6.90)	53 (6.46)
	Maximum score (%)	77	75

^a^MET: metabolic equivalent of task.

^b^QoL: quality of life (measured using the short form version of the QoL Enjoyment and Satisfaction Questionnaire).

We performed descriptive analyses of the data. The data were presented using percentages, means, and SDs. As this was a feasibility study, no hypothesis testing was performed to assess the efficacy of the intervention [62]. Mean differences between baseline and follow-up were presented, accompanied by 95% CIs [62].

## Results

### Recruitment and Retention

**Figure 3:**
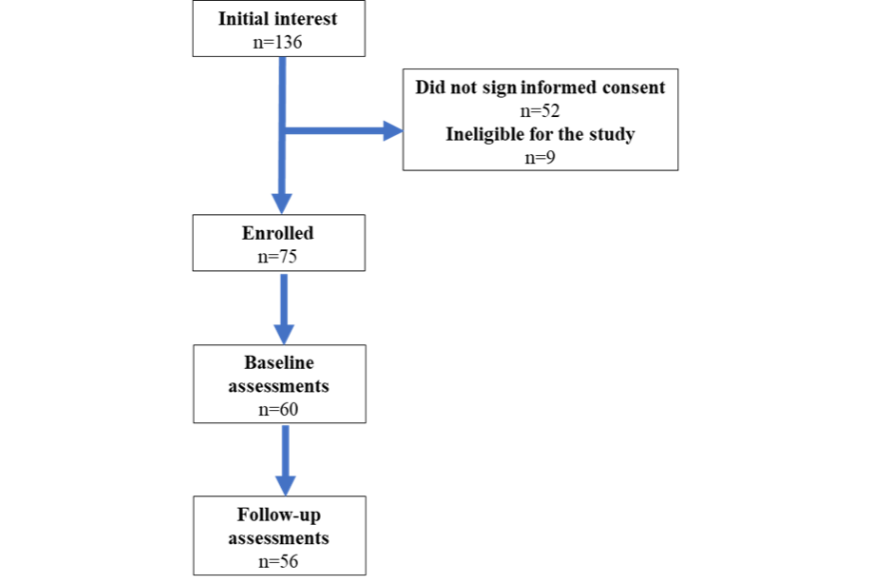
The flow of participants through the study.

A total of 136 individuals expressed initial interest in participating in the study; however, 9 (6.6%) were ineligible because of comorbidities or because they had not installed Facebook Messenger on their smartphone, and a further 52 (38.2%) completed the screening questionnaire but did not proceed to provide informed consent, leaving 75 (55.1%) participants eligible for participation. Of these 75 participants, 60 (80%) completed baseline assessments, and of these 60, 56 (93%) completed the follow-up questionnaires (Figure 3).

### Characteristics of Participants

Of the enrolled participants, 38% (23/60) were men. The mean age was 33.7 years (SD 9.3), the mean BMI was 22.3 kg/m^2^ (SD 3.8; Table 2), and the average technical competency on a scale of 1 to 10 was 8.07.

**Table 2:** Characteristics of all the enrolled participants who completed baseline assessments (N=60).

Characteristics			Baseline values
Age (years), mean (SD)			33.7 (9.3)
BMI (kg/m^2^), mean (SD)			22.3 (3.8)
Gender (male), n (%)			23 (38)
**Ethnicity, n (%)**			
	Chinese		48 (80)
	Malay		5 (8)
	Indian		3 (5)
	**Others**		
		White	2 (3)
		Burmese	2 (3)
**Marital status, n (%)**			
	Currently married		25 (42)
	Never married		33 (55)
	Separated		1 (2)
	Divorced		1 (2)
**Highest level of education, n (%)**			
	University and above		50 (83)
	Polytechnic diploma		1 (2)
	Other diploma and professional qualification		1 (2)
	A^a^-level or NTC^b^-1 or NTC-2 or certificate in office or business skills or its equivalent		6 (10)
	O^a^ or N^c^-level or NTC-3 certificate or its equivalent		1 (2)
	Secondary school		1 (2)
**Work status, n (%)**			
	Employed		37 (62)
	Student (full time)		17 (28)
	Homemaker or housewife		3 (5)
	Unemployed (able to work)		2 (3)
	Retired		1 (2)
**History of parents, sibling, or child with type 2 diabetes, n (%)**			
	Yes		11 (18)
	No		49 (82)
**History of hypertension, n (%)**			
	Yes		2 (3)
	No		58 (97)

^a^A-level or advanced-level examinations are taken by students at the age of 18, 2 or 3 years after completing their O-level or ordinary-level examinations, which are taken by students at the age of 16 after 4 years (or 5 years) of secondary school.

^b^NTC: National Technical Certificate.

^c^N-level: The Singapore-Cambridge General Certificate of Education Normal-level. Secondary students in Singapore can move between two streams based on their academic performance: 4 years of study culminating in the O-level (ordinary level) or the N-level (normal level) examinations. N-level students may participate in a fifth year of study to take the O-level examinations.

### Satisfaction

Of the 56 participants who followed the study through till completion, 52 (92%) were moderately or very satisfied with Precilla, 30 (54%) thought that their likelihood of recommending Precilla to others would be *somewhat likely*, 32 (57%) felt that the likelihood of their opting to use Precilla again for personal use would also be *somewhat likely*, and, finally, 29 (51%) posited that their health was somewhat better or much better than before.

### Usability

**Figure 4:**
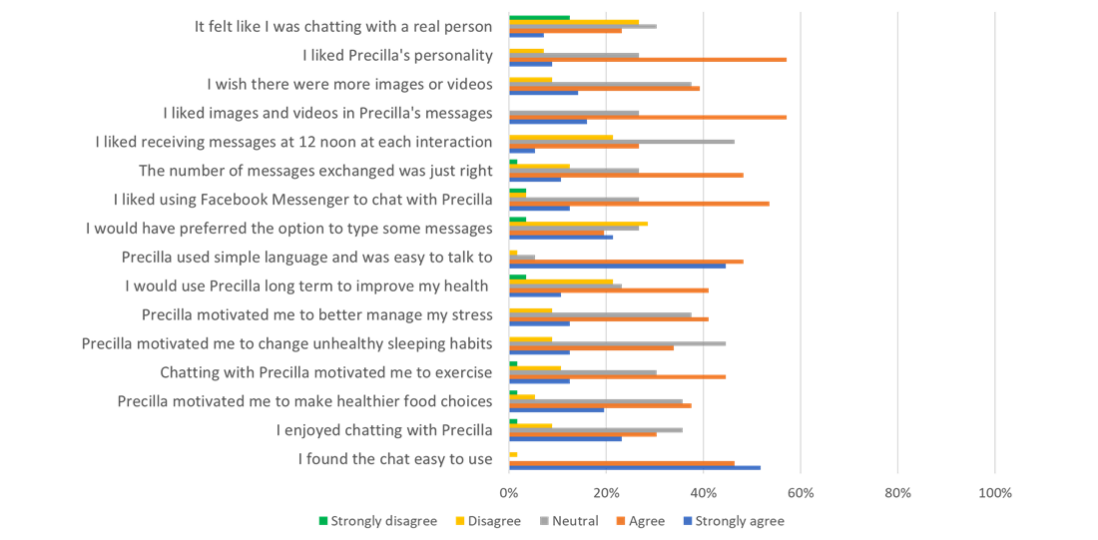
Participants’ opinions of Precilla’s usability and acceptability.

The *agree* and *strongly agree* responses exceeded 50% (28/56) for all the questions relating to Precilla’s acceptability, except for question 5, “Chatting with Precilla motivated me to change unhealthy sleeping habits,” where the collective response in agreement (agree and strongly agree) was only 46% (26/56) (Figure 4). The smallest percentage of disagreement was for questions 1 and 8, indicating that most of the participants (55/56, 98%) found the chat easy to use and thought that Precilla used simple language and was easy to talk to. The *neutral* response was a common selection by participants for questions 2-7.

Of the 56 participants, 37 (67%) agreed that Facebook Messenger was an appropriate medium to deliver the messages sent by Precilla. The remaining participants were either neutral or disagreed. *Telegram* and *WhatsApp* were proposed as potential alternative delivery media. Of the 56 participants, 9 (15%) thought that the number of messages exchanged with Precilla was too high, and they would have preferred the intervention to deliver fewer messages; 12 (21%) disagreed that 12 PM was the most appropriate time to receive messages, whereas the remaining 44 (79%) either agreed, strongly agreed, or were neutral (Figure 4) and indicated a preference for interacting with Precilla after work, in the evening or before bedtime. Some indicated specific timings such as 5 PM, 8 PM, or 10 PM, and 1 participant suggested 9 AM or during the morning commute to work. There were no disagreements to statement 13 (“I liked images and videos in Precilla’s messages”) and minimal disagreement for statements 14 (5/56, 9%) and 15 (4/56, 7%) on the inclusion of more visual components and Precilla’s personality, respectively. Most participants did not find Precilla to be very human-like and could clearly tell that they were communicating with a conversational agent. Of the 56 participants, 3 (5%) suggested some degree of personalization, whereby Precilla’s messages should be initiated when the user prompted the conversational agent and not the other way around.

### Conversational Agent Engagement Data

**Figure 5:**
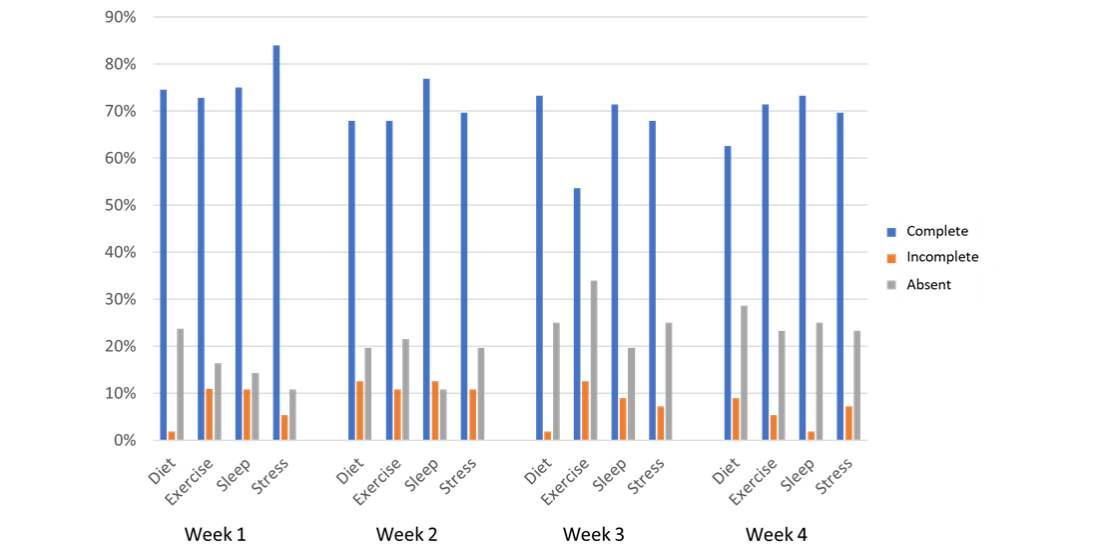
Percentage of total weekly interactions that were complete, incomplete, or absent (not attempted at all).

**Figure 6:**
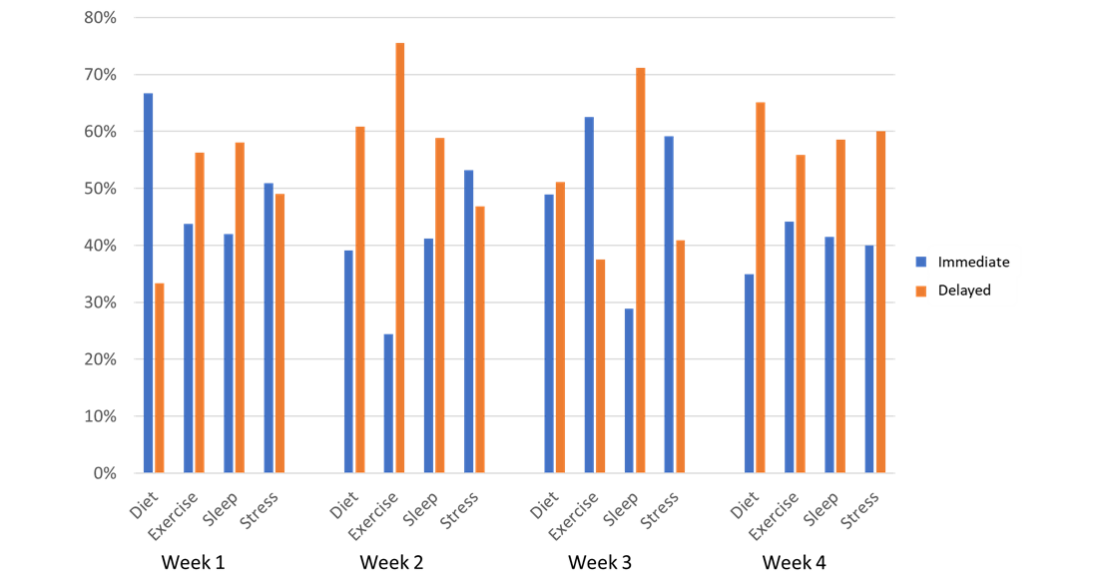
Number of immediate and delayed interactions between participants and Precilla for each category.

The number of complete, incomplete, and absent interactions were noted (Figure 5). In addition, the number of immediate and delayed interactions were also counted (Figure 6). Some interactions were started but not completed. The reasons for not completing interactions are presented in Multimedia Appendix 4.

All the conversations were completed by at least 50% (>30/56) of the participants consistently over the period of the study. The number of participants who completed interactions from start to finish exceeded 71% (40/56) for 56% (9/16) of the interactions, denoting high engagement with the conversational agent. Similarly, at least 40% (<22/56) of the participants responded immediately to the conversational agent 75% (12/16 interactions) of the time, even in the last week of the study, denoting consistently high conversational agent engagement.

The participants were restricted to a predetermined list of options when providing responses to the questions asked or information provided. A frequent cause of disruption to the conversation flow was participants typing free text, which led them off track. This resulted in their being redirected to the main menu, which they potentially found confusing, or they were reluctant to go through the whole conversation flow again and, hence, failed to complete that interaction. The average duration of each interaction was in the 2- to 5-minute range. More data on conversational agent engagement are presented in Multimedia Appendix 4.

### QoL Score

The 14-item QoL score was 54 (SD 6.90) at baseline and 53 (SD 6.46) at follow-up (mean difference 0.95; 95% CI −1.51 to 3.41). Of the participants taking medication (10 at baseline, 17 at follow up), participants rated adherence as fair (baseline 5/10, 50%; follow-up 6/17, 35%), good (baseline 5/10, 50%; follow-up 9/17, 53%), and very good (baseline 0/10, 0%; follow-up 2/17, 12%). The participants rated overall life satisfaction as fair (baseline 16/60, 27%; follow-up 12/56, 21%), good (baseline 35/60, 58%; follow-up 33/56, 59%), or very good (baseline 9/60, 15%; follow-up 10/56, 18%), and 2% (1/56) of participants reported poor overall life satisfaction at follow-up.

### Knowledge

The scoring system for the questionnaires meant that lower scores denoted better knowledge outcomes (sections 1 and 3) and lower perceived risk of diabetes (section 2). In section 1, the scores were 7 (SD 1.37) at baseline and 6 (SD 0.93) at follow-up (mean difference 0.52, 95% CI 0.09-0.95); the possible score range was 6-18. In section 2, the scores were 28 (SD 2.7) at baseline and 27 (SD 3.5) at follow-up (mean difference 0.9, 95% CI −0.25 to 2.05); the possible score range was 12-48. In section 3, the scores were 10 (SD 4.11) at baseline and 9 (SD 2.2) at follow-up (mean difference 1.0, 95% CI −0.23 to 2.23); the possible score range was 8-32.

### Diet

The number of individuals consuming vegetables at least once a day was 27% (16/60) at baseline and 29% (16/56) at follow-up. Similarly, the percentage of participants having three portions of fruit per day was 3% (2/60) at baseline and 7% (4/56) at follow-up. With regard to intake of unhealthy food comprising sweetened beverages and fried food or snacks, the *almost never* consumption category for sweetened beverages was 38% (23/60) at baseline and 45% (25/56) at follow-up, whereas for fried food and snacks, the *almost never* consumption category was 25% (15/60) at baseline and 30% (17/56) at follow-up.

### Physical Activity

The median METs-per-week score was 857 at baseline (IQR 902) and 765 (IQR 882) at follow-up. The average categorical score remained constant from baseline to follow-up where the average exercise intensity was *moderate*. The mean time spent sitting was 439 minutes at baseline and 406 minutes at follow-up. In addition, the median amount of moderate and vigorous physical activity was the same, 30 min per week (IQR 90) at baseline and 50 min per week (IQR 90) at follow-up.

### Sleep

According to the scoring method, a lower score indicated better sleep. The mean PSQI score was 4.38 (SD 2.36) at baseline and 4.43 (SD 2.45) at follow-up (mean difference −0.05, 95% CI −0.93 to 0.83). The prevalence of poor sleep quality was 28% (16/60) at baseline and 30% (16/56) at follow-up. The participants who reported sleeping for less than 7 hours were classified as short sleepers; 82% (49/60) were short sleepers at baseline, whereas 88% (49/56) were short sleepers at follow-up.

### Stress

The scoring for stress meant that lower scores denoted lower stress levels and higher scores denoted higher stress levels. The mean score at baseline was 17 (SD 5.13), denoting a moderate stress level, which was maintained even at follow-up, mean 16 (SD 5.1; mean difference 0.73; 95% CI −1.15 to 2.61).

## Discussion

### Principal Findings

To our knowledge, this is the first web-based pilot study to test the feasibility and acceptability of a healthy lifestyle behavior change conversational agent in the Singaporean population. In this web-based study, which used Facebook Messenger as a delivery medium for a conversational agent targeting healthy lifestyle behavior change in the Singaporean population, we managed to recruit 37.5% (75/200) of the target sample size in 1 month. Retention was high at 93% (56/60); conversational agent engagement was also high, with all the conversations being completed by at least 50% (28/56) of the participants consistently. Furthermore, at least 40% (22/56) of the participants responded almost immediately 75% (12/16) of the time. Acceptability, usability, and satisfaction were also generally high. In general, we were able to conduct the study with high fidelity, and each phase ensued as planned. Any definitive signs of improvement in QoL, knowledge, diet, physical activity, sleep, and stress would have to be studied in more detail in a future study of effectiveness.

The secondary outcomes in the study were measured using validated scales, namely, the short form version of the QoL Enjoyment and Satisfaction Questionnaire, PSQI, Perceived Stress Scale, Food Frequency Questionnaire, and IPAQ. We took note that although these questionnaires have not necessarily been optimized for the Singaporean population, these well-established scales were chosen to ensure a certain level of validity and reliability in our results.

### Comparisons With Existing Literature

Conversational agents have been used for healthy behavior change; however, they have been used mainly in niche areas such as smoking cessation, alcohol misuse treatment, and the promotion of physical activity in sedentary populations [63-65]. The application presented in this study is very comprehensive, novel, and relevant to the general population in Singapore.

The outcome measures were similar to those in other studies looking at the acceptability of conversational agents in health care, and acceptability was high in other studies as well as in this feasibility trial. Previous studies noted high response rates (ie, conversational agent engagement) and strongly agree or agree scores for user-friendliness, appropriateness, consistency, and speed of response, as in our study [66]. The other measures in prior studies that denoted acceptability were high perceived ease of use, usefulness, and intention to use, similar to our measures of ease and enjoyment of use, motivating change in unhealthy habits, and intention to use Precilla again or recommend it to others, respectively [67,68].

In another study using a health behavior change conversational agent, high compliance was attributed to a rewarding game system [68]. Considering the slight decrease in weekly compliance in our feasibility study (43/56, 77% completed interactions in week 1 vs 39/56, 69% in week 4), it may be worth exploring the possibility of introducing a gaming or reward component in future iterations. Personalization has previously been met with positive user reception, for example, by providing personalized advice to maintain target blood glucose levels or personalized reminders for taking medication [69]. Similarly, personalized content and message timing delivery should be implemented in future versions of Precilla, considering the preference indicated by users.

It has previously been indicated that users tend to prefer interacting with female conversational agents, as evidenced by Siri, Cortana, and Alexa, for example [70]. In addition, the study by Brahnam et al [71] has explained that in the field of human-computer interaction, “the standard of believability has become inextricably linked to gender personification, especially female personification.” As such, we chose to use *Precilla* and reinforced her character with a profile picture.

Views on conversational agent personality have been mixed, depending on the function of the agent. For example, participants appreciated an empathetic demeanor from conversational agents for e-therapy, in contrast to the participants’ indifference to Precilla’s humanity in this pilot study, where she played more of an impersonal, informative or educational role [72]. It may be that the conversational agent’s purpose determines how important the degree of humanity is.

Another aspect of this study was the effectiveness of participant recruitment through social media. We chose a goal of 75% (150/200) recruitment rate based on other studies involving mobile health trials having achieved an application rate of 86% (70/81) for enrollment through Facebook or an 85.1% (605/711) response rate to Facebook advertisements in 45 days [73,74]. Although our study only yielded a recruitment rate of 37.5% (75/200) in the given period, it aligns quite appropriately with the recruitment rates of 33%, 30%, or 37.7% in other web-based mobile apps or Facebook trials [75-77]. Hence, our 37.5% (75/200) recruitment rate seems to indicate feasibility regarding the recruitment of participants in the general population in Singapore for a web-based conversational agent intervention delivered through Facebook.

A systematic review accentuated the effectiveness of Facebook as a recruitment tool [78]. It was suggested that on average, studies allowed a 3-month recruitment period, and Facebook was found to be more efficient than traditional methods (print, radio, television, email, or word of mouth) because of the reduced costs, shorter recruitment times, and ability to connect with harder-to-reach populations. In future studies, we may need to consider complementing our current no-fee Facebook approach with advertisements or other recruitment methods, as well as potentially a longer recruitment period.

### Implications and Future Research

In terms of the recruitment, we noticed some attrition among the eligible participants just before provision of informed consent. As the only communication was through email from an institutional email address, the uncertainty involved in remote participation, such as never meeting the study team in person, could have been a cause for the participants’ apprehension. This lack of direct engagement may have led to some participants not feeling comfortable sharing their digital signature with us because of data protection and privacy concerns. In future research studies, a mix of a digital recruitment approach and face-to-face exchange (possibly in the form of recruitment or debriefing) could provide participants with an opportunity to ask questions and validate the legitimacy of the study.

Regarding the preliminary efficacy, minimal improvements were observed in the participants’ knowledge, stress, and diet, whereas there was a lack of improvement in QoL and sleep and no change in physical activity. The dearth of significant improvement in these areas could be attributed to the content and delivery not being adequately designed to target effectiveness and, possibly, the short study length (4 weeks), which may not have been sufficient for any noteworthy changes to have been observed. Furthermore, this study was not designed to test the effectiveness of the intervention, and this should be explored in more detail in future iterations.

The participants indicated that they could clearly tell that Precilla was indeed a conversational agent. This was beneficial in confirming Precilla’s identity and in reducing expectations from users that the conversational agent should think, function, and react like a human being. It was interesting to note that although the participants did not find Precilla to be very human-like, they still rated the conversational agent highly in terms of content, usability, general acceptability, ease of use, visual components, and so on. This shows that, for this study population and for a conversational agent with Precilla’s functions and capabilities, acceptability was not dependent on the anthropomorphism of the conversational agent.

The conversational agent engagement data revealed that there was a slight decrease in the number of interactions that the participants completed fully with each successive week. It is possible that the content and delivery methods could have come across as repetitive because we provided a review of take-home points from the previous interaction from week 2 onwards. Alternatively, perhaps the routine that Precilla followed made the intervention very regimented, not leaving much room for spontaneity in the form of varied message timings or even variations in the ways that users could respond to Precilla, for example, using free text. These factors may have contributed to the decrease in interest over time. Future iterations of Precilla could explore introducing more novelty and personalization, as recommended by the participants in the telephone interviews. The participants shared some points for improvement, including alternative modes of delivery such as WhatsApp and Telegram, a possibility of shortening the conversation lengths, and more personalized timings for message delivery.

### Strengths and Limitations

One of the main strengths of the study is the high fidelity in the delivery of this low-cost, fully web-based feasibility study. We did not experience any software malfunctions and were able to implement the intervention as per protocol. The content of the intervention was evidence based, and it was co-designed with members of the target population to make the intervention as relevant to them as possible. We managed to reach a diverse range of age groups—from 23 to 60 years—using Facebook without the need to pay for advertising. Although the recruited sample—recruited over 4 weeks using solely Facebook—consisted of fewer participants than our target size, it is comparable with those reported in other studies that used paid Facebook advertisements within the same time frame. However, the participants were recruited through healthy living–focused Facebook groups and pages; therefore, these individuals were already very much invested in healthy lifestyle behavior change and more likely to use apps and social media. Furthermore, the participants were Facebook users, capable of navigating Facebook Messenger, had high technological competency, and a very high level of tertiary education. As such, our findings may not be generalizable to other demographic groups such as older people; individuals with lower education levels; or those not active on, or familiar with, social media. However, this study did largely cover the general characteristics of the average Singaporean, making the findings very much valuable and relevant. A further limitation is that although we used validated outcome measurement tools, all outcomes were self-reported by the participants. Furthermore, although these measurement scales are well reputed and established, they were not necessarily optimized for use in the Singaporean population. Finally, we used a single-arm pre-post study design, which is acceptable for a feasibility study but does not allow for assessment of the effectiveness of the intervention.

### Conclusions

This web-based feasibility study showed that the delivery of a conversational agent for healthy lifestyle behavior change using Facebook Messenger is, to a large extent, feasible in Singapore. Precilla is a low-cost intervention that was popular among the participants and was well received, with most participants being satisfied with the intervention and prepared to recommend it to friends and family. This study demonstrated the ability to conduct a web-based trial to assess the impact of a novel intervention. Our preliminary data on the acceptability of the intervention showed the need for further enhancement of this conversational agent intervention, potentially through humanization of the agent and personalization of the messaging. Such an intervention needs to be evaluated with a rigorous study design and larger sample size.

## Appendix

Multimedia Appendix 1 Sources of information for the conversational agent.

Multimedia Appendix 2 Dialogue tree sample outline for introductory week. The message blocks are in grey, and answer button options in blue. User’s name will be seen in the {first name} area. The “typing” lasts between 2-5 seconds depending on the length of text to be read.

Multimedia Appendix 3 Questionnaires administered to participants.

Multimedia Appendix 4 Conversational agent engagement data.

## Abbreviations

IPAQ: International Physical Activity Questionnaire
MET: metabolic equivalent of task
PSQI: Pittsburgh Sleep Quality Index
QoL: quality of life
REDCap: Research Electronic Data Capture
