# Supplementary material for: Conversational Agent for Healthy Lifestyle Behavior Change: Web-Based Feasibility Study
Source: JMIR Form Res. 2021 Dec 3;5(12):e27956. doi: 10.2196/27956 (PMC8686401; doi:10.2196/27956)
Supplement: Multimedia Appendix 1 [file formative_v5i12e27956_app1.docx]

**Multimedia Appendix 1.** Sources of information for the conversational agent

| **Conversational agent domain** | **Weekly components derived** | **Sources** |
| --- | --- | --- |
| Prediabetes & diabetes | First week introduction | - War on diabetes: Singapore to host global conference on tackling epidemic - What is pre-diabetes - Prediabetes - Prevention of type 2 diabetes mellitus by changes in lifestyle among subjects with impaired glucose tolerance - Lean diabetes mellitus: An emerging entity in the era of obesity |
| Diet | Week 1: Fibre  Week 2: Proteins & Fats  Week 3: Recap week 1  Week 4: Recap week 2 | - Walnut Consumption Is Associated with Lower Risk of Type 2 Diabetes in Women - Dairy Products and Prevention of Type 2 Diabetes: Implications for Research and Practice - Popcorn is more satiating than potato chips in normal-weight adults - Fruit and vegetable intake and incidence of type 2 diabetes mellitus: systematic review and meta-analysis - Intake of Fruit, Vegetables, and Fruit Juices and Risk of Diabetes in Women  The role of diet in the prevention of type 2 diabetes |
| Sleep | Week 1: Bedtime routine  Week 2: Stimulus control  Week 3: Recap and knowledge recall of week 1  Week 4: Recap and knowledge recall of week 2 | - Sleepless no more: Techniques and Interventions for Sleep Disorders - The effects of improving sleep on mental health (OASIS): a randomised controlled trial with mediation analysis - Can music improve sleep quality in adults with primary insomnia? A systematic review and network meta-analysis - Insomnia: Relaxation techniques and sleeping habits  Too early to get up, too late to get back to sleepThe evidence base of sleep restriction therapy for treating insomnia disorder |
| Exercise | Week 1: Aerobic & Anaerobic activity  Week 2: Exercise options matched to preferences  Week 3: Recap and knowledge recall of week 1  Week 4: Recap of exercise options to choose from (week 2) | - Health hub SG: Aim for 150 minutes of physical activity every week  Physical Activity/Exercise and Diabetes: A Position Statement of the American Diabetes AssociationExercise for diabetes and get a leg upBenefits of exercise and physical activityReversing Diabetes Through Weight Loss: How Much Should You Lose?Different types of physical activity |
| Stress | Week 1: Deep breathing  Week 2: Mindfulness  Week 3: Recall week 1 & relaxing music  Week 4: Mindfulness recap | - Stress Management Apps With Regard to Emotion-Focused Coping and Behavior Change Techniques: A Content Analysis - Managing and Controlling Stress Using mHealth: Systematic Search in App Stores  Stress Management: Breathing Exercises for Relaxation  - Getting started with Mindfulness - Stress Management Techniques: evidence-based procedures that reduce stress and promote health |
