# Supplementary material for: Conversational Agent for Healthy Lifestyle Behavior Change: Web-Based Feasibility Study
Source: JMIR Form Res. 2021 Dec 3;5(12):e27956. doi: 10.2196/27956 (PMC8686401; doi:10.2196/27956)

**Multimedia appendix 2.** Dialogue tree sample outline for introductory week. The message blocks are in grey, and answer button options in blue. User’s name will be seen in the {first name} area. The “typing” lasts between 2-5 seconds depending on the length of text to be read.


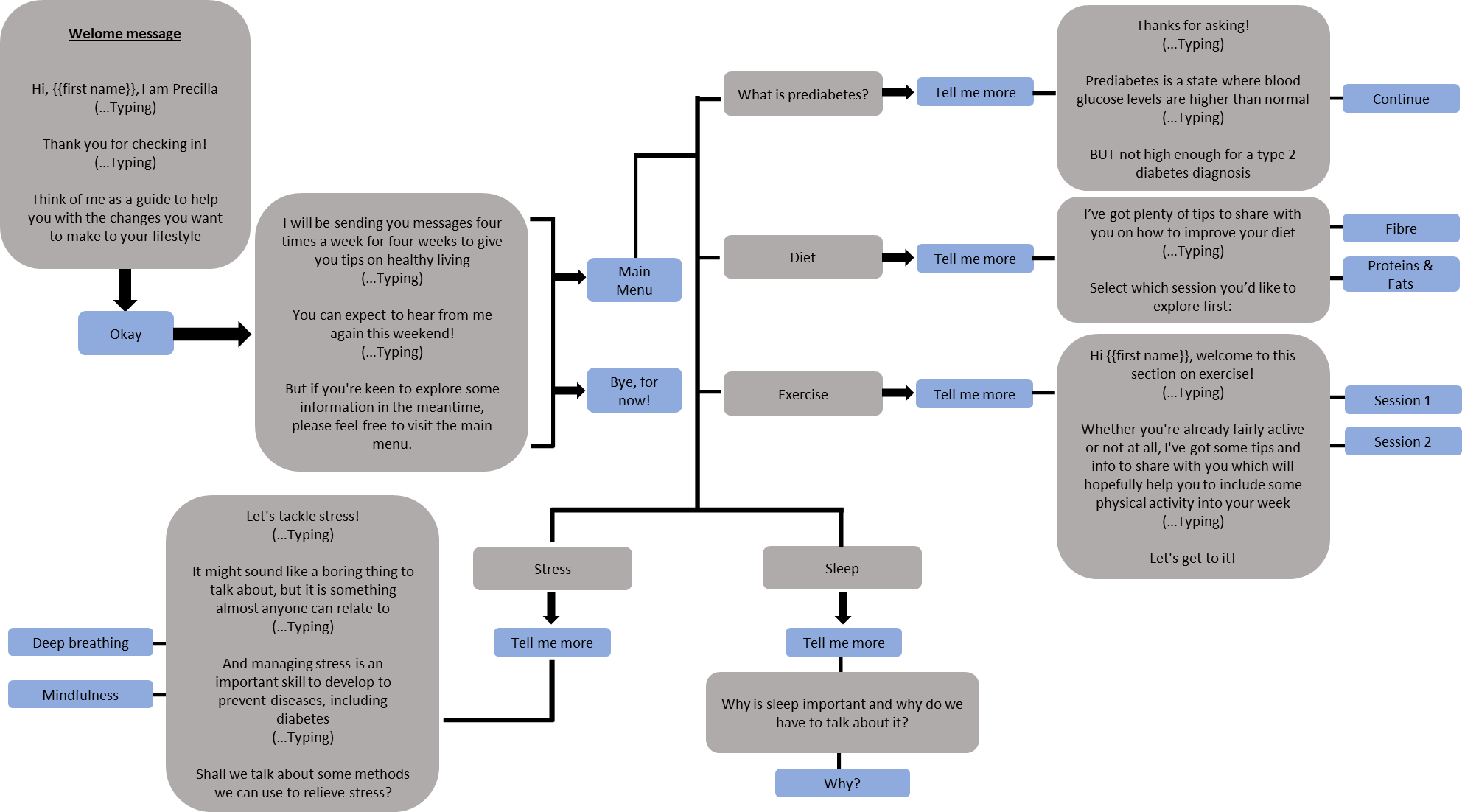

Supplement: Multimedia Appendix 2 [file formative_v5i12e27956_app2.docx]
