# Supplementary material for: Conversational Agent for Healthy Lifestyle Behavior Change: Web-Based Feasibility Study
Source: JMIR Form Res. 2021 Dec 3;5(12):e27956. doi: 10.2196/27956 (PMC8686401; doi:10.2196/27956)
Supplement: Multimedia Appendix 3 [file formative_v5i12e27956_app3.docx]

**Multimedia Appendix** 3. Questionnaires

**Quality of life (QOL) questionnaire**

**Table 1.** 14 item quality of life questionnaire. Each item was rated on a 5-point scale.

| **Taking everything into consideration, during the past week how would you rate your .....** | Work | Family relationships | Sexual drive, interest and/or performance | ability to get around physically without feeling dizzy or unsteady or falling |
| --- | --- | --- | --- | --- |
| Physical health | Household activities | Leisure time activities | Economic status | ability to do work or hobbies |
| mood | Social relationships | Ability to function | Living/housing situation | overall sense of well being |

**Knowledge questionnaire**

**Table 2.** Knowledge questionnaire section 1.

| For each item below, let us know the response that **BEST DESCRIBES YOUR OPINION** of diabetes and prediabetes | **Scoring for Q1-6:**  Yes = 1  No = 2  I don’t know = 3  **Q7 answer options:**  Almost no chance, slight chance, moderate chance, high chance |
| --- | --- |
| Q1 | Diabetes is a disease that occurs when your blood sugar is too high |
| Q2 | Prediabetes is a condition in which someone's blood sugar levels are higher than normal but not high enough to be diagnosed a diabetic |
| Q3 | People with diabetes have a higher chance of getting diabetes |
| Q4 | Regular exercise may help prevent diabetes from developing |
| Q5 | A healthy diet may help prevent diabetes from developing |
| Q6 | Weight loss may help prevent diabetes from developing |
| Q7 | What do you think your risk or chance is for getting diabetes over the next 10 years? |

**Table 3.** Knowledge questionnaire section 2

| **What statement best reflects your opinion for each?** | **Scoring**:  Strongly agree = 1  Strongly disagree = 4 |
| --- | --- |
| Q1. I feel that I have little control over risks to my health | Q7. Over the past year I made conscious food choices to improve my diet |
| Q2. If I am going to get diabetes, there is not much I can do about it | Q8. Over the past year I chose to be active to improve my health |
| Q3. I think that my personal efforts will help control my risks of getting diabetes | Q9. Over the past year I lost weight through conscious efforts (ie. Not due to sickness or stress) |
| Q4. People who make a good effort to control the risks of getting diabetes are much less likely to get diabetes | Q10. In the coming year I will make food choices to improve my diet |
| Q5. Compared to other people of my same age, I am less likely that they are to get diabetes | Q11. In the coming year I chose to be active to improve my health |
| Q6. Compared to other people of my same age, I am less likely that they are to get a serious disease | Q12. In the coming year I plan to lose weight |

**Table 4.** Knowledge questionnaire section 3

| We would like you to think about members of the general public here. Check the box for each statement that best describes your opinion. | Scoring:  Increases or raises risk = 1  Has no effect on risk = 2  Decreases or lowers the risk = 3  Don’t know = 4 |
| --- | --- |
| Q1. Having a sibling or a parent with diabetes | Q5. Having high blood pressure |
| Q2. Being 40 years old and above | Q6. Having abnormal blood cholesterol or lipid levels |
| Q3. Being overweight or obese | Q7. Having a history of gestational diabetes |
| Q4. Leading an inactive lifestyle | Q8. Having prediabetes |

**Diet – Food frequency questionnaire**

**The next couple of questions will be about your diet.**

**We would like to know how often you consume each of the following food items per day. Please select the option which best represents your daily intake of 1 serving of the following food and drinks.**

Vegetables

- Almost never
- Once
- Twice
- Three times
- More than 3 times

(same options below)

Fruit (exclude juices)

Sugar-free sweetened beverages

Fried food/snacks

**Physical activity Questionnaire**

1. **In the last 7 days, can you please indicate how many days you spent doing:**

- vigorous physical activity like heavy lifting, aerobics, fast bicycling?
- moderate intensity activity like carrying light loads, regular paced cycling, doubles tennis?
- low intensity physical activity like walking?
- 0 days
- 1-3 days
- 5-7 days

1. **How much time did you usually spend doing:**

- vigorous physical activities on one of those days?
- moderate intensity physical activity on one of those days?
- 10-20 mins per session
- 20-30 mins per session
- 30-60 mins per session
- >60 mins per session

1. **If you were physically active, could you please indicate the length of each exercise session? (in minutes)**

- 10-20 mins per session
- 20-30 mins per session
- 30-60 mins per session
- >60 mins per session

**During the last 7 days, how much time (in hours per day) did you spend sitting on a weekday?**

[Short answer text]

**Stress Questionnaire**

The questions in this scale ask you about your feelings, thoughts and stress levels during the last month. Please do feel free to be honest. Your responses will be kept confidential amongst the study team members, please do not worry about your data being shared publicly.

For each question, please indicate how often you felt or thought a certain way in the past month.

Been upset because of something that happened unexpectedly?

- Never
- Almost never
- Sometimes
- Fairly often
- Very often

(same options throughout)

Felt that you were unable to control the important things in your life?

Felt nervous and "stressed"?

Felt confident about your ability to handle your personal problems?

Felt that things were going your way?

Felt that you could not cope with all the things you had to do?

Been able to control irritations in your life?

Felt that you were on top of things?

Been angered because of things that were outside of your control?

Felt difficulties were piling up so high that you could not overcome them?

**Sleep Questionnaire**

The following questions relate to your usual sleep habits during the past month only. Your answers should indicate the most accurate reply for the majority of days and nights in the past month.

1. **What time have you usually gone to bed at night? (Please use 12 hr times with am/pm (eg. 10.30pm)**

[Short answer text]

1. **How long has it usually taken you to fall asleep each night?**

- <30 mins
- 30-60 mins
- 1-2 hrs
- >2 hrs

1. **What time have you usually gotten up in the morning? (Please use 12 hr times with am/pm (eg. 9.15am)**

[Short answer text]

1. **How many hours of actual sleep did you get at night on weekdays?**

- <6 hrs
- 6-7 hours
- 8 hours
- >8 hours

1. **How many hours of actual sleep did you get at night on weekends?**

- <6 hrs
- 6-7 hours
- 8 hours
- >8 hours

1. **During the past month, how often have you had trouble sleeping?**

- Yes
- No

1. **(if yes above) How often have you had trouble sleeping?**

- Not during the past month
- Less than once a week
- Once or twice a week
- Three or more times a week

1. **Could you please select from the list provided, the potential reasons for your sleeping problems? You may choose multiple answers.**

- I Cannot get to sleep within 30 minutes
- I Wake up in the middle of the night or early morning
- I Get up to use the bathroom
- I Cannot breathe comfortably
- I Cough or snore loudly
- I Feel too cold
- I Feel too hot
- I Had bad dreams
- I Have pain
- Other

1. **If you have reasons besides those stated, please describe it here**

**(short answer text)**

1. **During the past month, how would you rate your sleep quality overall?**

- Very bad
- Bad
- Decent
- Good
- Very good

**How often have you taken medicine to help you sleep (prescribed or over the counter)?**

- Not during the past month
- Less than once a week
- Once or twice a week
- Three or more times a week

**How often have you had trouble staying awake while driving, eating meals, or engaging in social activity?**

- Not during the past month
- Less than once a week
- Once or twice a week
- Three or more times a week

**How much of a problem has it been for you to keep up enough enthusiasm to get things done?**

- No problem at all
- Only a very slight problem
- Somewhat of a problem
- A very big problem

**Satisfaction questionnaire**

The four specific questions were:

Q1 - How would you rate your overall satisfaction with the healthy lifestyle program delivered by Precilla?

(Answer options: Very satisfied, moderately satisfied, dissatisfied)

Q2 - How likely are you to recommend "Precilla" to others?

(Answer options: Extremely likely, highly likely, somewhat likely, unlikely, very unlikely)

Q3 - In the future if a healthy lifestyle program is available through Precilla, how likely would you want to use Precilla again?

(Answer options: Extremely likely, highly likely, somewhat likely, unlikely, very unlikely)

Q4 - As a result of the help you received from Precilla, would you say your health is…

(Answer options: neither better nor worse/somewhat better than before/much better than before.)
