# Supplementary material for: Conversational Agent for Healthy Lifestyle Behavior Change: Web-Based Feasibility Study
Source: JMIR Form Res. 2021 Dec 3;5(12):e27956. doi: 10.2196/27956 (PMC8686401; doi:10.2196/27956)
Supplement: Multimedia Appendix 4 [file formative_v5i12e27956_app4.docx]

**Multimedia appendix 4 – Conversational agent engagement data**

|  | **No. of completed interactions** | **No. of immediate interactions** | **No. of delayed interactions** | **Average duration of each interaction (mins)** | **Number of incomplete interactions** | **Number of absent interactions** | **Total** (complete + incomplete + absent) | **Reasons for incomplete**  **interactions (if any)** |
| --- | --- | --- | --- | --- | --- | --- | --- | --- |
| **Week 1** |  |  |  |  |  |  |  |  |
| Diet | 41 | 28 | 14 | 2.25 | 1 | 13 | 55 | (1) free text (can you tell me more?),  (2) possible tech difficulty. “Fibre” flow appeared twice. Left and returned to convo 30 mins later, (3) possible tech difficulty. Multiple repetitions of same block. Keep pressing the button? |
| Exercise | 40 | 21 | 27 | 3.54 | 6 | 9 | 55 | (1) free texting (eg. *Can I please redirect you to the main menu?* That's fine, no need. *Are you ready to get started?* Sure. *do you exercise?* |
| Sleep | 42 | 21 | 29 | 3.67 | 6 | 8 | 56 | (1) Detour to main menu caused by free text entry |
| Stress | 47 | 27 | 26 | 2.59 | 3 | 6 | 56 | (1) possible tech difficulties |
| **Week 2** |  |  |  |  |  |  |  |  |
| Diet | 38 | 18 | 28 | 1.97 | 7 | 11 | 56 | (1) Free text = conversation detoured to main menu, (2) possible tech difficulties |
| Exercise | 38 | 11 | 34 | 4.11 | 6 | 12 | 56 | (1) free text = conversation detoured to main menu |
| Sleep | 43 | 21 | 30 | 2.44 | 7 | 6 | 56 | (1) possible tech difficulties. Multiple pressing of the same button. |
| Stress | 39 | 25 | 22 | 1.97 | 6 | 11 | 56 | (1) Possible tech difficulties. Same button recurring. (2) Free text = conversation detoured to main menu |
| **Week 3** |  |  |  |  |  |  |  |  |
| Diet | 41 | 22 | 23 | 2.10 | 1 | 14 | 56 | NA |
| Exercise | 30 | 25 | 15 | 4.00 | 7 | 19 | 56 | Possible tech difficulties? Same button recurring. |
| Sleep | 40 | 13 | 32 | 1.80 | 5 | 11 | 56 | (1) free text (thumbs up) conversation detoured to main menu and halted |
| Stress | 38 | 26 | 18 | 2.11 | 4 | 14 | 56 | NA |
| **Week 4** |  |  |  |  |  |  |  |  |
| Diet | 35 | 15 | 28 | 2.34 | 5 | 16 | 56 | NA |
| Exercise | 40 | 19 | 24 | 3.15 | 3 | 13 | 56 | (1) free text "*grapeseed.*  *Not rapeseed*". Conversation halted at this point. |
| Sleep | 41 | 17 | 24 | 1.56 | 1 | 14 | 56 | NA |
| Stress | 39 | 18 | 27 | 2.26 | 4 | 13 | 56 | NA |
